# Fibrinogen-Induced *Streptococcus mutans* Biofilm Formation and Adherence to Endothelial Cells

**DOI:** 10.1155/2013/431465

**Published:** 2013-10-08

**Authors:** Telma Blanca Lombardo Bedran, Jabrane Azelmat, Denise Palomari Spolidorio, Daniel Grenier

**Affiliations:** ^1^Department of Oral Diagnosis and Surgery, Araraquara Dental School, State University of São Paulo, 14801-903 Araraquara, SP, Brazil; ^2^Oral Ecology Research Group, Faculty of Dentistry, Laval University, 2420 Rue de la Terrasse, Quebec City, QC, Canada G1V 0A6; ^3^Department of Physiology and Pathology, Araraquara Dental School, State University of São Paulo, 14801-903 Araraquara, SP, Brazil

## Abstract

*Streptococcus mutans*, the predominant bacterial species associated with dental caries, can enter the bloodstream and cause infective endocarditis. The aim of this study was to investigate *S. mutans* biofilm formation and adherence to endothelial cells induced by human fibrinogen. The putative mechanism by which biofilm formation is induced as well as the impact of fibrinogen on *S. mutans* resistance to penicillin was also evaluated. Bovine plasma dose dependently induced biofilm formation by *S. mutans*. Of the various plasma proteins tested, only fibrinogen promoted the formation of biofilm in a dose-dependent manner. Scanning electron microscopy observations revealed the presence of complex aggregates of bacterial cells firmly attached to the polystyrene support. *S. mutans* in biofilms induced by the presence of fibrinogen was markedly resistant to the bactericidal effect of penicillin. Fibrinogen also significantly increased the adherence of *S. mutans* to endothelial cells. Neither *S. mutans* cells nor culture supernatants converted fibrinogen into fibrin. However, fibrinogen is specifically bound to the cell surface of *S. mutans* and may act as a bridging molecule to mediate biofilm formation. In conclusion, our study identified a new mechanism promoting *S. mutans* biofilm formation and adherence to endothelial cells which may contribute to infective endocarditis.

## 1. Introduction

*Streptococcus mutans* is one of the most important etiologic agents of dental caries [1, 2]. It efficiently colonizes the oral cavity because of its ability to form biofilms on dental surfaces. When *S. mutans* is grown in the presence of sucrose, it produces glucans and fructans that contribute to the formation of a dense, adherent biofilm that allows acids to accumulate on the tooth surface, resulting in enamel demineralization [3]. *S. mutans *can also form biofilms by a sucrose-independent mechanism [4].

Under certain circumstances (dental procedures, oral infections, dental hygiene, and eating), *S. mutans *can gain access to the bloodstream, thus causing a transient bacteremia [5]. Nakano et al. recently reported that *S. mutans *was the oral bacterial species most frequently detected in cardiovascular specimens, suggesting that it enters the bloodstream more readily than other oral bacteria [6]. While, in most cases, the bacteremia has no serious consequences, *S. mutans* can adapt to this environment, resist to the immune defense, colonize the heart, and cause infective endocarditis, a severe and often fatal systemic disease [7]. Individuals with congenital cardiac malformations, with prosthetic aortic valves or having a compromised immune system, are more susceptible to develop infective endocarditis [7]. *S. mutans* is commonly isolated in cases of infective endocarditis, which is a typical biofilm-associated infection [8]. Bacteria colonizing the host in biofilms are more resistant to the host immune defense and to killing by antibiotics in comparison with planktonic bacteria [9]. 

The ability of *S. mutans* to colonize the cardiac valves and form biofilms is critical for its capacity to cause infective endocarditis. Recently, Jung et al. [10] brought *in vitro *and *in vivo* evidence indicating that host factors can modulate biofilm formation in *S. mutans*. More specifically, they showed that interactions between platelets and *S. mutans *play a key role in biofilm formation [10]. It is likely that additional host factors also contribute to promote the formation of biofilm by *S. mutans*. The aim of this study was to investigate *S. mutans* biofilm formation and adherence to endothelial cells induced by human fibrinogen. The putative mechanism by which biofilm formation is induced and the impact of fibrinogen on the resistance to penicillin of* S. mutans* were also evaluated.

## 2. Materials and Methods

### 2.1. Bacteria and Growth Conditions

Eight strains of *S. mutans* (ATCC 25175, ATCC 31383, ATCC 35668, IFN, NY257, UA96, 12A, and 33A) were used in this study. The bacteria were grown aerobically at 37°C in Todd-Hewitt broth (BBL Microbiology Systems, Cockeysville, MD, USA) supplemented with hemin (10 *μ*g mL^−1^) and vitamin K (10 *μ*g mL^−1^) (THB-HK).

### 2.2. Effects of Plasma and Various Plasma Proteins on Biofilm Formation

The ability of *S. mutans *to form biofilms when grown in the presence of bovine plasma (Sigma-Aldrich Canada Co., Oakville, ON, Canada; 100%, 50%, 25%, and 12.5% in 10 mM phosphate-buffered saline (PBS, pH 7.2)) was tested in 96-well polystyrene tissue culture plates. The effect of various plasma proteins (5 mg mL^−1^), including human fibrinogen (EMD Millipore, Billerica, MA, USA), human *γ*-globulin (Sigma-Aldrich Canada Co.), bovine serum albumin (BSA) (Fisher Scientific Company, Ottawa, ON, Canada), and human transferrin (Sigma-Aldrich Canada Co.), on biofilm formation by *S. mutans *was evaluated in a similar fashion. The effect of twofold serial dilutions of human fibrinogen (5 to 0.039 mg mL^−1^) in THB-HK medium was also tested. Lastly, biofilm formation in THB-HK medium with and without fibrinogen (5 mg mL^−1^) and supplemented with twofold serial dilutions of sucrose (1% to 0.0156%) was tested. An overnight culture of *S. mutans* was diluted in fresh THB-HK medium to get an optical density at 655 nm (OD_655_) of 0.2 (10^8^ colony forming units (cfu) mL^−1^). Samples (100 *μ*L) were added to the wells of a 96-well polystyrene tissue culture plate containing 100 *μ*L of THB-HK medium ± the protein described above. After a 48 h incubation at 37°C, the OD_655_ was measured to estimate bacterial growth. Culture medium and free-floating bacteria were then removed by aspiration, the wells were washed twice with PBS, and the biofilms were stained with 0.05% crystal violet dye (100 *μ*L) for 10 min. The wells were washed twice with PBS to remove unbound crystal violet dye and were dried for 2 h at 37°C. After adding 100 *μ*L of 95% (v/v) ethanol to each well, the plate was shaken for 10 min to release the stain from the biofilms, and the absorbance at 550 nm (*A*_550_) was determined to quantify biofilm formation. Uninoculated wells containing culture medium served as controls.

### 2.3. Scanning Electron Microscopy

The structural architecture of *S. mutans* biofilms formed in the presence of fibrinogen (0.156 mg mL^−1^), plasma (50%), or sucrose (0.25%) was observed by scanning electron microscopy. Briefly, *S. mutans *suspended at a final OD_655_ of 0.1 in plasma or THB-HK containing one of the test compounds was added (2 mL) to the wells of a 6-well polystyrene tissue culture plate, each well containing a 13 mm diameter plastic coverslip (Nunc, Kastrup, Denmark). The culture plate was incubated at 37°C for 48 h. The culture medium and free-floating bacteria were then removed by aspiration. The biofilm-coated coverslips were incubated overnight at 4°C in fixation buffer (4% paraformaldehyde, 2.5% glutaraldehyde, and 2 mM CaCl_2_ in 0.2 M cacodylate buffer, pH 7.2), washed twice with 0.1 M cacodylate buffer (pH 7.0), and postfixed for 90 min at room temperature in 1% osmic acid containing 2 mM potassium ferrocyanide and 6% sucrose in 0.1 M cacodylate buffer (pH 7.0). The biofilms on the coverslips were dehydrated using a graded series of ethanol (50%, 70%, 95%, and 100%), critical point dried, gold sputtered, and examined using an electron microscope (JEOL JSM6360LV; JEOL, Tokyo, Japan) operating at 15 kV.

### 2.4. Determination of Minimal Inhibitory and Minimal Bactericidal Concentrations of Penicillin

The minimal inhibitory (MIC) and minimal bactericidal concentrations (MBC) of penicillin for *S. mutans *grown in THB-HK were determined using a microbroth dilution method. The wells of a 96-well microplate, each well containing 100 *μ*L of serially diluted penicillin (penicillin G; 20–0.039 *μ*g mL^−1^ in THB-HK), were inoculated with 100 *μ*L of an overnight culture of *S. mutans* diluted in fresh culture broth to obtain an OD_655_ of 0.2 (10^8^ CFU mL^−1^). The MIC was the lowest concentration of antibiotic for which no significant increase in OD_655_ was noted after a 24 h incubation at 37°C. To determine the MBC, 10 *μ*L of culture was collected from wells with no apparent growth and spread on THB-HK agar plates. The MBC was the lowest concentration of antibiotic at which no colonies grew on the plates after 48 h of incubation at 37°C. The MIC and MBC for biofilm-grown *S. mutans* were determined using a microplate containing a 24 h preformed fibrinogen-induced biofilm in each well. The culture supernatants were aspirated, and 200 *μ*L of twofold serial dilutions of penicillin in fresh culture broth was added to the wells. The plate was incubated at 37°C for 24 h. The biofilm-grown bacteria were then suspended by scraping the bottom of the wells with a 200 *μ*L pipet tip and then pumping the solution up and down until a homogenous suspension was obtained. Growth was then estimated by recording the OD_655_. The MIC was the lowest concentrations of penicillin for which no significant increase in OD_655_ was noted. The MBC of the biofilm-grown cells was determined by spreading 10 *μ*L of the resuspended biofilm on a THB-HK agar plate. The MBC was the lowest concentration of antibiotic for which no colonies grew on the agar medium after 48 h of incubation.

### 2.5. Effect of Fibrinogen on the Adherence of *S. mutans* to Endothelial Cells

The effect of fibrinogen on the adherence of fluorescein isothiocyanate (FITC)-labeled *S. mutans *to human endothelial cells was evaluated. A 10 mL aliquot of a 24 h culture of *S. mutans* was centrifuged (7000 ×g for 10 min), and the pellet was suspended in 12 mL of 0.5 M NaHCO_3_ (pH 8) containing 0.03 mg mL^−1^ FITC. The bacterial suspension was incubated in the dark at 37°C for 30 min with constant shaking. The bacteria were then washed three times by centrifugation (7000 ×g for 5 min) and were suspended in the original volume of PBS. Immortalized human brain microvascular endothelial cells kindly provided by Dr. Marcelo Gottschalk (Université de Montréal, QC, Canada) were grown in RPMI-1640 medium (HyClone, Logan, UT, USA) supplemented with 10% heat-inactivated FBS, 10% Nu-serum IV supplement (BD Biosciences, Bedford, MA, USA), and 50 *μ*g mL^−1^ of penicillin-streptomycin, until they reached confluence, as previously described [11]. The cells were harvested by gentle trypsinization with 0.05% trypsin-ethylenediaminetetraacetic acid (Invitrogen, Grand Island, NY, USA) at 37°C and were suspended in RPMI-1640 medium (without FBS and Nu-serum). Aliquots of cell suspension (100 *μ*L, 1.5 × 10^6^ cells mL^−1^) were placed in the wells of 96-well black plates (Greiner Bio-One, St. Louis, MO, USA). After a 4 h incubation at 37°C in a 5% CO_2_ atmosphere to allow a confluent monolayer to form, spent medium was aspirated, 100 *μ*L of 1% glutaraldehyde was added to the wells, and the plate was incubated at 4°C overnight. Glutaraldehyde was aspirated, and the wells were washed three times with PBS. Filtered 1% bovine serum albumin (100 *μ*L) was added to each well, and the plate was incubated for 30 min at 37°C. The wells were washed once with PBS, 100 *μ*L of fibrinogen (final concentration: 0, 0.01, 0.1, 1, and 10 mg mL^−1^) was added to each well, and the plate was incubated for 30 min. FITC-labeled *S. mutans* cells were then added (100 *μ*L) to the wells at a multiplicity of infection (MOI) of 200, and the plate was incubated in the dark for 2 h at 37°C. Unbound bacteria were removed by aspiration, and the wells were washed three times with PBS. Relative fluorescence units (RFU; excitation wavelength 495 nm; emission wavelength 525 nm) corresponding to the level of bacterial adherence were determined using a microplate reader. Control wells without fibrinogen were used to determine the 100% adherence value. Wells containing only endothelial cells and fibrinogen were also prepared to determine the autofluorescence values related to fibrinogen. 

### 2.6. Assay for Fibrinogen Conversion into Fibrin

The ability of *S. mutans* (cells and culture supernatant) to convert fibrinogen into fibrin was assayed using a plate assay, as previously described [12]. Briefly, a solution containing 1.2% agarose and 0.4% human fibrinogen was prepared in 100 mM tris-HCl buffer (pH 7.4), and 10 mL was poured into a 100 × 15 mm Petri dish. After solidification, wells (7 mm diameter) were cut in the agarose and were filled with 120 *μ*L of *S. mutans* cell suspension (OD = 1.0) or culture supernatant. Thrombin (0.4 unit) was used as a positive control and tris-HCl (pH 7.4) was used as a negative control. After a 5 h incubation at 37°C, the Petri dish was examined. The presence of an opaque zone around a well indicated that the fibrinogen had been converted into fibrin. 

### 2.7. Assay for Fibrinogen-Binding Activity

The fibrinogen-binding activity of *S. mutans *cells was investigated using human fibrinogen-Alexa Fluor conjugate (Invitrogen). Briefly, bacteria from an overnight culture of *S. mutans *were harvested by centrifugation and were suspended in PBS containing fluorescent fibrinogen (40 *μ*g/mL). The mixture was incubated in the dark at room temperature for 60 min. The bacteria were then washed three times with PBS by centrifugation and were suspended in the original volume of PBS. Aliquots of bacterial suspension (100 *μ*L) were placed in the wells of 96-well black plates (Greiner Bio-One). RFU (excitation wavelength 495 nm; emission wavelength 525 nm) corresponding to fibrinogen bound to *S. mutans* were determined using a microplate reader and were compared to a standard curve generated using fluorescent fibrinogen at different concentrations. As a control, *S. mutans* cells were preincubated with nonfluorescent fibrinogen (0.5, 0.25, and 0.15 mg mL^−1^) for 1 h at room temperature prior to incubating them in the presence of fibrinogen-Alexa Fluor conjugate. 

### 2.8. Statistical Analysis

All the experiments were run in triplicate in two independent experiments, and the means ± standard deviations (SD) were calculated. The statistical analysis was performed using Students *t*-test.

## 3. Results

**Figure 1:**
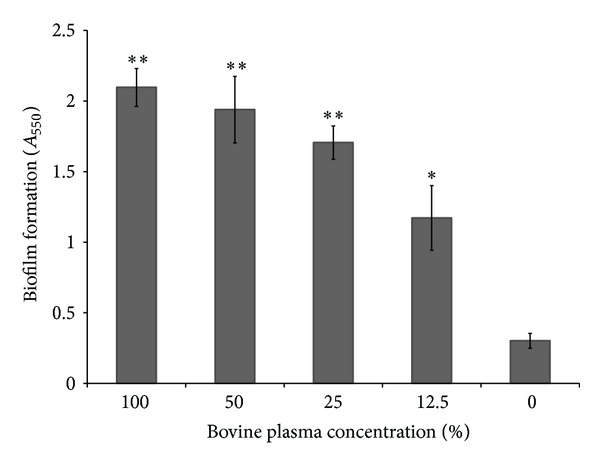
Biofilm formation by *S. mutans *following growth in bovine plasma as quantified by crystal violet staining. Results are expressed as means ± standard deviations of triplicate assays from two independent experiments. *P < 0.01 and **P < 0.001: significantly different from the control without bovine plasma (growth in THB-HK medium).

**Figure 2:**
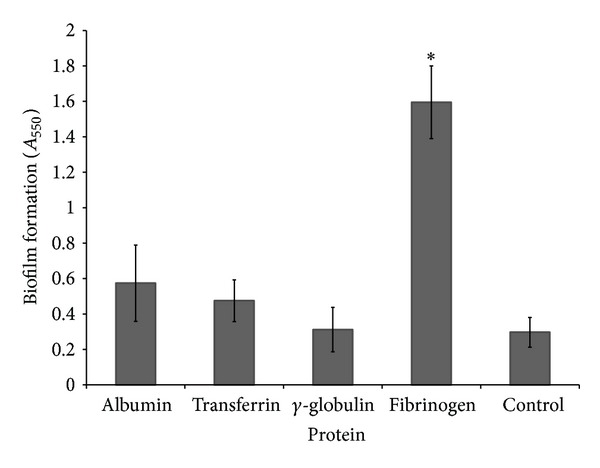
Effect of various plasma proteins added to THB-HK medium on biofilm formation by *S. mutans*. Results are expressed as means ± standard deviations of triplicate assays from two independent experiments. *P < 0.001: significantly different from the control (without plasma proteins).

**Figure 3:**
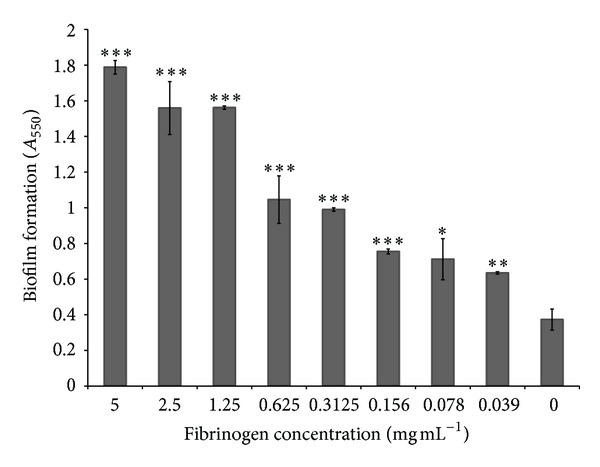
Effect of various concentrations of human fibrinogen added to THB-HK medium on biofilm formation by *S. mutans*. Results are expressed as means ± standard deviations of triplicate assays from two independent experiments. *P < 0.05, **P < 0.01, and ***P < 0.001: significantly different from the control (without fibrinogen).

*S. mutans* (ATCC 25175) formed a more significant biofilm when grown in bovine plasma than in THB-HK (Figure 1). In undiluted and 12.5% (v/v) plasma, the amount of biofilm increased 7- and 4-fold, respectively, compared to the biofilm formed in THB-HK. To identify the plasma component that induced biofilm formation, *S. mutans* (ATCC 25175) was grown in THB-HK supplemented with various plasma proteins. Fibrinogen promoted the formation of biofilm, while albumin, transferrin, and *γ*-globulin had no significant effect (Figure 2). Moreover, fibrinogen dose dependently induced biofilm formation by *S. mutans*. At the highest concentration of fibrinogen tested (5 mg mL^−1^), the amount of biofilm formed was approximately 5-fold higher than that formed in unsupplemented culture medium (Figure 3). Even at the lowest concentration tested (0.039 mg mL^−1^), fibrinogen had a significant effect on biofilm formation. Fibrinogen-induced biofilm formation was not related to a growth-promoting effect since the final OD_655_ was the same in all the assays (data not shown). To determine whether the above phenomenon was strain specific, additional strains of *S. mutans *were tested. As reported in Table 1, the fibrinogen-induced biofilm formation was observed for five out of eight strains of *S. mutans*. Further analyses were carried out with strain ATCC 25175 (serotype c).

**Figure 4:**
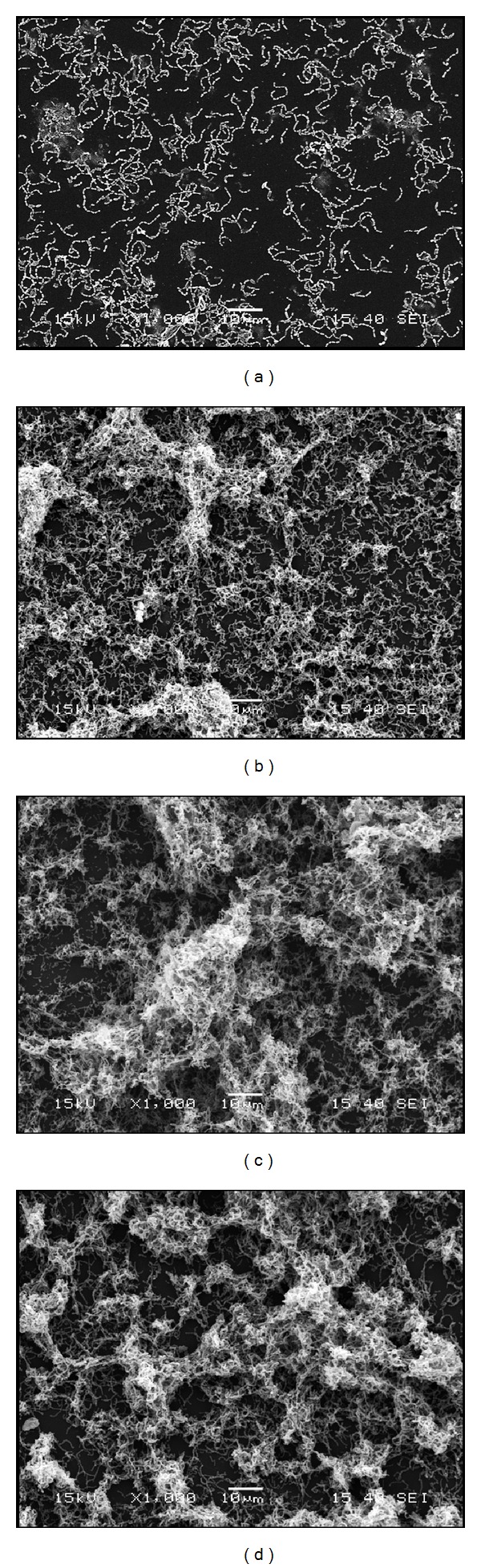
Scanning electron micrographs of *S. mutans *biofilms formed on plastic tissue culture coverslips following growth in THB-HK medium (a), 50% bovine plasma (b), THB-HK medium + fibrinogen (0.156 mg mL^−1^) (c), and THB-HK medium + sucrose (0.25%) (d). Magnification 1000x.

The structural architecture of the *S. mutans* biofilm formed in the absence and presence of human fibrinogen was examined by scanning electron microscopy. In the absence of fibrinogen, short chains of *S. mutans* were observed attached to the polystyrene surface and were rarely bound to each other (Figure 4(a)). However, when the culture medium was supplemented with fibrinogen (0.156 mg mL^−1^), aggregates and microcolonies of *S. mutans* completely covered the surface of the support (Figure 4(c)). Similar results were observed when *S. mutans *was grown in the presence of 50% plasma (Figure 4(b)) and 0.25% sucrose (Figure 4(d)).

**Figure 5:**
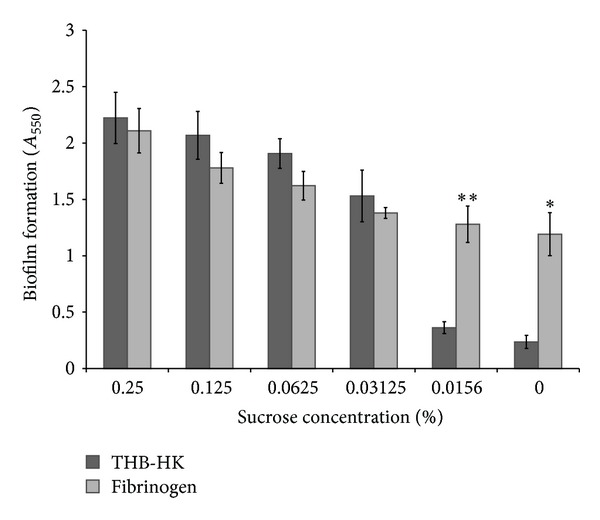
Effect of various concentrations of sucrose added to THB-HK medium in the presence and absence of fibrinogen on biofilm formation by *S. mutans*. Results are expressed as means ± standard deviations of triplicate assays from two independent experiments. *P < 0.01 and **P < 0.001: significantly different from the control (without fibrinogen).

We then tested whether sucrose and fibrinogen in combination had an additive effect on biofilm formation by *S. mutans*. As expected, sucrose (starting at 0.031%) had a dose-dependent effect on the amount of biofilm formed (Figure 5). However, no additive effect was observed in the presence of both sucrose and fibrinogen.

The MIC and MBC of penicillin G for *S. mutans* grown in THB-HK (planktonic form) were 0.0195 and 1.25 *μ*g mL^−1^, respectively. The MIC and MBC for biofilm-grown *S. mutans* were also determined using preformed fibrinogen-induced biofilms. In this case, the MIC was the same as that for the planktonic form of *S. mutans*, while the MBC was 5 *μ*g mL^−1^. 

**Figure 6:**
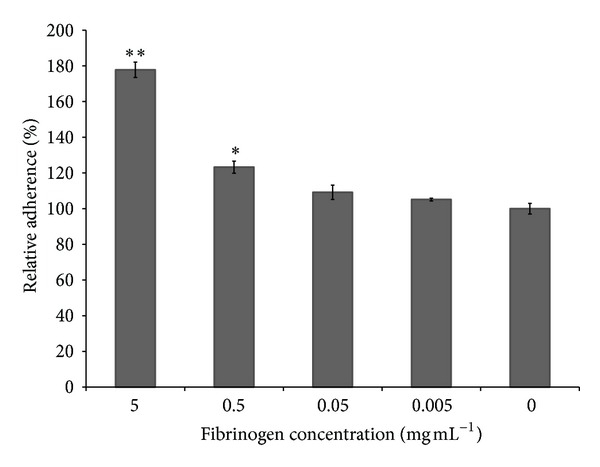
Effects of fibrinogen on the adherence of *S. mutans* to human endothelial cells. Results are expressed as means ± standard deviations of triplicate assays from two independent experiments. A value of 100% was assigned to the control (without fibrinogen). *P < 0.05 and **P < 0.01: significantly different from the control (without fibrinogen).

We then evaluated the capacity of fibrinogen to promote the adherence of *S. mutans* to endothelial cells. Fibrinogen dose dependently increased bacterial adherence to endothelial cells (Figure 6). At the highest concentration tested (5 mg mL^−1^), fibrinogen increased the adherence of *S. mutans* to endothelial cells by 75%. However, no significant differences were observed at the lowest concentrations tested (0.005 and 0.05 mg mL^−1^).

**Figure 7:**
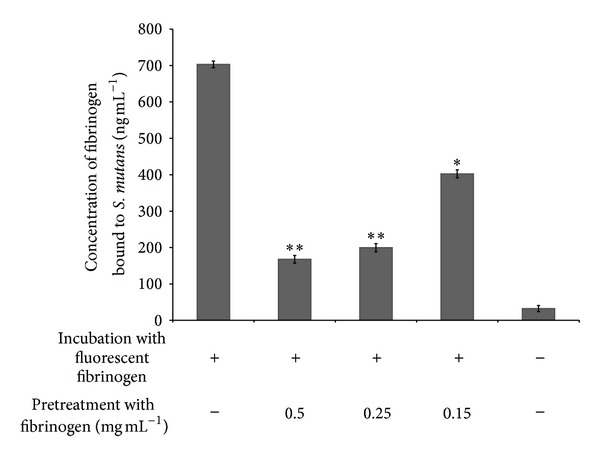
Effect of fibrinogen on the binding of fibrinogen-Alexa Fluor conjugate to *S. mutans* cells. *P < 0.01: significantly different from the control (without nonfluorescent fibrinogen).

The potential mechanisms by which human fibrinogen promotes *S. mutans* biofilm formation and adherence were then investigated. First, the ability of *S. mutans* (cells and culture supernatant) to convert fibrinogen into fibrin was assayed using a plate assay. Neither *S. mutans* cells nor culture supernatants converted fibrinogen into fibrin, while the positive control (thrombin) produced an opaque zone (data not shown). We then evaluated the capacity of *S. mutans *to bind fibrinogen to its cell surface, a phenomenon that may result in bridging and thus contributes to biofilm formation. *S. mutans* cells bound fibrinogen-Alexa Fluor conjugate (Figure 7). Pretreating *S. mutans* cells with nonfluorescent fibrinogen prior to incubating them with fibrinogen-Alexa Fluor conjugate resulted in a significant inhibition of fluorescence, suggesting that the binding is specific.

## 4. Discussion

The ability of bacteria to form biofilms on host surfaces is a crucial virulence factor and protects them against innate host defenses and antimicrobial agents. The formation of biofilms is important in the pathogenesis of several subacute and chronic human bacterial infections, including endocarditis [13]. Bacterial endocarditis is frequently caused by commensal streptococci such as *S. mutans *that colonizes the cardiac valve in a biofilm composed of bacteria and their extracellular products, as well as host components (platelets, fibrin) [13]. In the present study, we showed that *S. mutans* biofilm formation on a polystyrene surface is promoted by plasma and that fibrinogen is the component responsible for this effect. Fibrinogen is a 340 kDa glycoprotein in human blood plasma that is involved in blood coagulation through its conversion into fibrin by thrombin [14]. The concentrations of fibrinogen required to induce biofilm formation by *S. mutans *are relevant to *in vivo* situations since the normal levels of fibrinogen in plasma are in the range of 1 to 4.5 mg mL^−1^. Jung et al. [10] previously reported that platelets are required for *S. mutans *biofilm formation in plasma, while our study showed that fibrinogen alone is sufficient. This discrepancy may be related to the bacterial strain used since we showed that fibrinogen-induced biofilm formation is not observed for all isolates of *S. mutans*. 

In our study, *S. mutans* growing in a fibrinogen-induced biofilm was found to be more resistant to the bactericidal activity of penicillin. This enhanced resistance to penicillin was likely related to the stable architecture of the biofilm, which restricted the penetration of the antibiotic. The presence of persister cells (dormant bacteria) may also explain the resistance of *S. mutans *in biofilms to killing by penicillin, which acts on growing cells.

Fibrinogen also increased the adherence of *S. mutans *to endothelial cells. Since *S. mutans *is known to invade human coronary artery endothelial cells [15] and that close interactions between bacteria and host cells are critical in the invasive process, this fibrinogen-induced adhesion may enhance the ability of *S. mutans *to enter endothelial cells and avoid being eliminated by the immune system. Interestingly, Cheung et al. reported that fibrinogen can act as a bridging molecule to promote the adherence of *Staphylococcus aureus *to cultured endothelial cells [16]. 

To identify the mechanism by which fibrinogen promotes biofilm formation by *S. mutans*, we evaluated the ability of this species to convert fibrinogen into fibrin and to bind fibrinogen to its cell surface. While *S. mutans* could not mediate the conversion of fibrinogen into fibrin, it possessed a fibrinogen-binding activity. This is in agreement with previous studies reporting that the major surface antigen I/II of *S. mutans *is involved in the fibrinogen-binding activity [17, 18]. This property may allow bacteria to attach to each other through fibrinogen-mediated crossbridging. Fibrinogen binding to streptococci also plays a significant role in enabling them to adhere to host surfaces and in protecting them from the host immune system, notably by preventing opsonophagocytosis [19, 20]. In a previous study, we showed that fibrinogen can specifically induce biofilm formation by *Streptococcus suis, *which is a major causative agent of infective endocarditis in swine [21]. As for *S. mutans*, *S. suis *can express fibrinogen surface receptors allowing fibrinogen to act as a bridging molecule. 

## 5. Conclusions

Our study identified a new mechanism promoting *S. mutans* biofilm formation and adherence to endothelial cells. Fibrinogen is specifically bound to the cell surface of *S. mutans* and may act as a bridging molecule. This phenomenon may contribute to infective endocarditis.

## Figures and Tables

**Table 1:** Effect of human fibrinogen (5 mg mL^−1^) added to THB-HK medium on biofilm formation by various strains of *S. mutans*. Results are expressed as means ± standard deviations of triplicate assays. *P < 0.01: significantly different from the control (without fibrinogen).

Strain	Biofilm formation (*A*_550_)	
	No fibrinogen	+Fibrinogen
ATCC 25175	0.322 ± 0.001	1.92 ± 0.026*
ATCC 31383	0.472 ± 0.05	1.436 ± 0.071*
ATCC 35668	0.777 ± 0.011	0.97 ± 0.055
IFN	0.691 ± 0.025	1.68 ± 0.065*
NY257	0.802 ± 0.113	2.006 ± 0.14*
UA96	0.659 ± 0.069	0.528 ± 0.098
12A	0.739 ± 0.005	1.814 ± 0.095*
33A	0.804 ± 0.063	1.01 ± 0.04
